# A measure of endosomal pH by flow cytometry in *Dictyostelium*

**DOI:** 10.1186/1756-0500-2-7

**Published:** 2009-01-12

**Authors:** Anna Marchetti, Emmanuelle Lelong, Pierre Cosson

**Affiliations:** 1 Dpt for Cell Physiology and Metabolism University of Geneva, Faculty of Medicine Centre Médical Universitaire, 1. rue Michel Servet, CH1211, Geneva 4, Switzerland

## Abstract

**Background:**

*Dictyostelium *amoebae are frequently used to study the organization and function of the endocytic pathway, and specific protocols are essential to measure the dynamics of endocytic compartments and their internal pH.

**Findings:**

We have revisited these classical protocols to measure more accurately endosomal pH, making use of a fluorescent probe (Oregon green) more adequate for very acidic pH values. This pH-sensitive probe was combined with a pH-insensitive marker, in order to visualize simultaneously endosome dynamics and pH changes. Finally, a flow cytometer was used to measure endosomal pH in individual cells.

**Conclusion:**

Using these simple protocols the endosomal pH of endocytic compartments can be assessed accurately, revealing the extreme acidity of *Dictyostelium *lysosomes (pH <3.5).

## Background

*Dictyostelium *amoebae are widely used to study the organization and function of the endocytic pathway. Due to their small, haploid, and fully sequenced genome [1], these cells are easily amenable to genetic studies. Analysis of specific knockout mutants has been instrumental in revealing the role of various gene products in diverse aspects of endocytosis, and this analysis relies on the use of well-documented protocols describing the various facets of endosome biology. In *Dictyostelium*, endocytosed material is found within minutes in lysosomes, which are acidified by the action of a V-type H^+^-ATPase [2]. Endocytosed material is then transferred more slowly (>30 minutes) to post-lysosomes devoid of H^+^-ATPase, which are less acidic [3,4]. The dynamics and organization of the endocytic pathway can be analyzed in a given mutant cell by determining the number and size of lysosomes and post-lysosomes, and the speed of transfer between these two compartments [5,6], or by measuring the pH of endosomal compartments.

In *Dictyostelium*, the endosomal pH has usually been measured by following the fluorescence of endocytosed fluorescein isothiocyanate (FITC)-coupled dextran in a spectrofluorimeter (reviewed in [7]). FITC has a pKa of approximately 6.5 and its ionization equilibrium leads to pH-dependent fluorescence over a range of 5 to 9. The values measured in early endocytic compartments (lysosomes) were as low as pH 4.7 [3,4], which corresponds to virtually total protonation of FITC. In an elegant study using ^31^P-NMR methods and aminomethylphosphonate (pKa 5.5), the lysosomal pH was found to be below 4.3, the lowest value that this probe could detect [8]. However this method is not adequate for common use, firstly because it relies on sophisticated equipment, and secondly because the probes used are eventually transferred to the cytosol, complicating analysis of the data. In this study, we used dextran coupled to Oregon-green, a probe with a low pKa (4.7) [9] to measure more accurately acidic lysosomal pH. In combination with a pH-insensitive fluorophore, this probe allowed us to follow at the same time fluid-phase kinetics (endocytosis, recycling) and pH variations. In addition, we used a flow cytometer to measure endosomal pH in individual cells, as previously reported in mammalian cells [10].

## Findings

### Methods

#### Cells and reagents

The subclone DH1-10 [11] of the DH1 *D. discoideum *axenic strain [12] was used in this study. An AX2 strain was also used in parallel experiments, with results virtually identical to the ones shown here (data not shown). Cells were grown and all experiments done at 21°C in HL5 medium: 14.3 g/L peptone (Oxoid LTD, Basingstoke, Hampshire, UK), 7.15 g/L yeast extract (Difco, Basel, Switzerland), 18 g/L maltose monohydrate (Sigma-Aldrich, Steinheim, Germany), 0.641 g/L Na_2_HPO_4 _dihydrate (Sigma-Aldrich), 0.490 g/L KH_2_PO_4 _(Sigma-Aldrich). Cells were passaged twice a week and maintained at a density below 10^6^cells/ml. Nigericin and monensin were obtained from Sigma-Aldrich.

#### pH measurement

To measure endosomal pH, cells were incubated in a shaken suspension (2 × 10^6 ^cells/ml) in HL5 medium containing FITC-coupled dextran (500 μg/ml, Invitrogen, Carlsbad, CA, USA), Oregon green-coupled dextran (250 μg/ml, Invitrogen) and/or Alexa647-coupled dextran (30 μg/ml, Invitrogen). After 20 minutes, cells were pelleted (Eppendorf centrifuge, 4000 rpm = 1500 g, 1 min), rinsed one time with HL5, and further incubated in HL5 medium. At the indicated time, an aliquot was used to measure cell-associated fluorescence in a FACSCalibur (Beckton Dickinson, San Jose, CA, USA). Cells were identified based on forward and side scatter. FITC and Oregon green fluorescence were measured in the FL1 channel (Excitation wavelength 488 nm; Emission 515–545 nm), Alexa647 was measured in the FL4 channel (Excitation 632 nm, Emission 655–695 nm). The median fluorescence value was determined for at least 5'000 cells. The background autofluorescence of cells not exposed to fluorescent dyes was determined and subtracted from every value.

#### Calibration curves

In each experiment, a calibration curve was obtained in parallel. For this, cells having endocytosed fluorescent dextrans for 20 minutes were washed and resuspended directly in ice-cold HL5 at the indicated pH (see below), supplemented with 40 mM NH_4_Cl (Merck KGaA, Darmstadt, Germany) and 0.1% (w/v) sodium azide (Merck) before FACS analysis. The equilibration of endosomal pH was almost immediate, and stable with time. The observed pKa of Oregon green was always within 0.5 pH units of the expected value (4.7).

To generate calibration curves, the pH of HL5 was adjusted with NaOH or HCl to the indicated values (6.7 in regular HL5). Since HL5 contains a phosphate buffer that is not ideal to obtain stable pH at acidic values, other buffers (MES-HL5, citrate-HL5, glycine-HL5) were also used (for pH values of 5–6–7, 4–5–6, 3–4, respectively), and identical calibration curves were obtained. For simplicity, phosphate-buffered HL5 was used in routine experiments.

## Results

### Calibration curves

**Figure 1:**
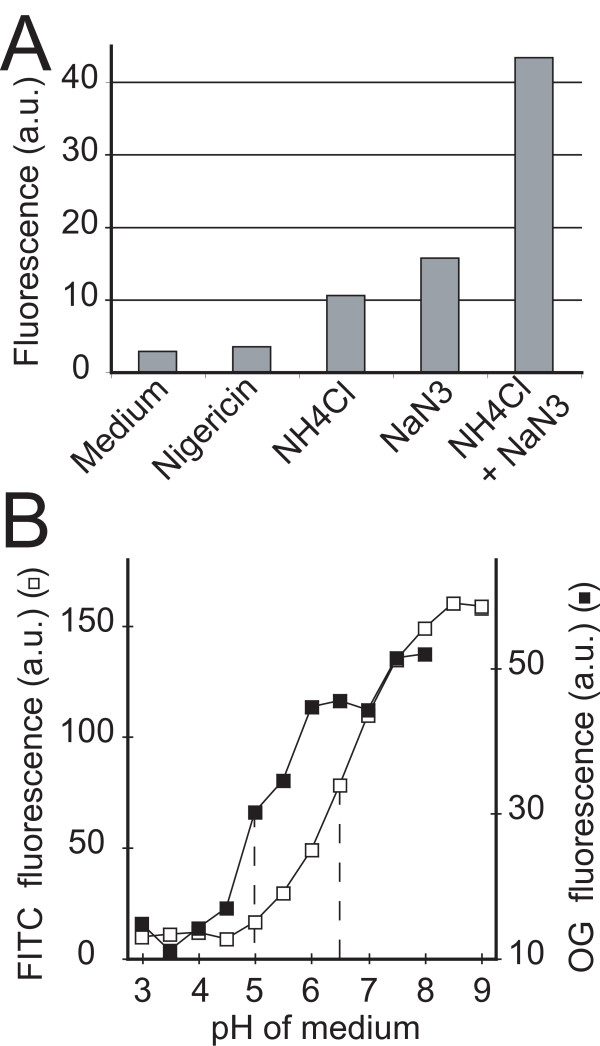
**pH calibration curves**. (A) A combination of ammonium chloride and of sodium azide allows neutralization of endosomal pH. To test the effect of various treatments on lysosomal pH, cells having internalized FITC-dextran were incubated at pH = 7.4 in medium alone (Medium), or in medium containing 10 μM nigericin (Nigericin), 40 mM ammonium chloride (NH_4_Cl), or 0.1% sodium azide (NaN_3_), as indicated. FITC fluorescence was then measured in a Fluorescence Activated Cell Sorter (FACSCalibur). Only sodium azide and ammonium choride increased lysosomal pH, as revealed by an increase in FITC fluorescence. The most pronounced effect was obtained in the presence of both sodium azide and NH_4_Cl. (B) Calibration curves of FITC and Oregon green fluorescence in *Dictyostelium *endosomes. *Dictyostelium *cells were allowed to internalize either FITC-dextran or Oregon green-dextran for 20 minutes. The cells were then washed and transferred to HL5 medium containing 0.1% sodium azide and 40 mM NH_4_Cl, at the indicated pH. The fluorescence was measured by flow cytometry. The approximate pKa values (5 for Oregon green and 6.5 for FITC) are close to the expected values, confirming that the external pH is effectively imposed in endosomal compartments in these conditions.

To generate calibration curves, one must be able to impose a defined pH in endosomal compartments in order to determine fluorescence emission at known pH values. To test various protocols, we allowed cells to internalize FITC-coupled dextran for 20 minutes. At this time, endocytosed fluid phase is found in acidic lysosomes. We then incubated the cells in neutral medium (pH 7.4), and tested the ability of various treatments to neutralize lysosomes and to increase fluorescein emission. Although it is often used in mammalian cells, in our hands nigericin (10 μM) did not significantly alter lysosomal pH (Fig. 1A), even in combination with monensin (5 μM) or KCl (100 mM) (data not shown). Sodium azide treatment (0.1%) as well as exposure to lysosomotropic NH_4_Cl (40 mM) increases lysosomal pH, and the maximal effect was obtained by associating these two drugs (Fig. 1A). Addition of a weak acid (40 mM acetate) did not modify these results (data not shown). In order to determine how efficient this treatment was at imposing the extracellular pH in lysosomes, cells having internalized either FITC-dextran or Oregon green-dextran were incubated in medium containing sodium azide and ammonium chloride at different pH. As expected, the fluorescence of FITC-dextran decreased at low pH values, and the observed pKa was close to the expected value of 6.5 (Fig. 1B). Similar results were obtained with Oregon green-dextran, and as expected, the observed pKa was close to 4.7 (Fig. 1B). These results indicate that the extracellular pH is imposed in lysosomes in the presence of azide and ammonium chloride, although minor differences between the extracellular and endosomal pH may persist.

### Kinetics of endosomal pH evolution

**Figure 2:**
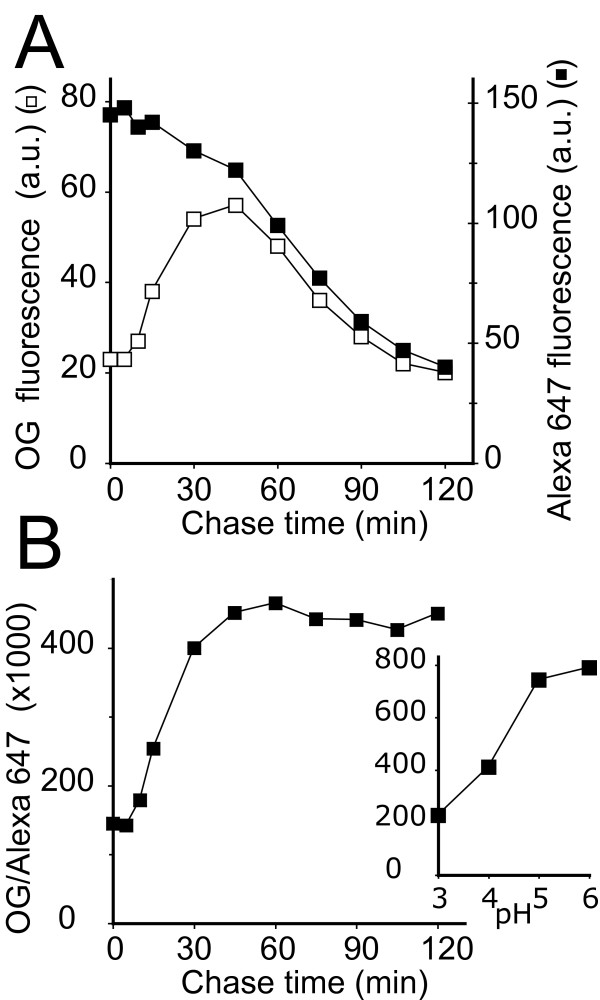
**Measurement of endosomal kinetics and pH**. (A) *Dictyostelium *cells were allowed to internalize for 20 minutes a mixture of Oregon green- and Alexa 647-dextran. The cells were then washed and further incubated in medium for the indicated time. At each time point, the cellular fluorescence was determined by flow cytometry. (B) To extrapolate endosomal pH values, the Oregon green/Alexa 647 ratio was calculated at each time point. A calibration curve was obtained in each individual experiment as described in Figure 1 (inset). These results indicate that early endocytic compartments (lysosomes) are very acidic, and that endocytosed fluid phase is then gradually transferred to less acidic compartments (post-lysosomes).

In order to analyze precisely the evolution of endosomal pH, we resorted to dual-fluorescence [13] by allowing cells to internalize both Oregon green-coupled dextran and Alexa 647-coupled dextran, the fluorescence of which is insensitive to pH (see Additional file 1). Oregon green and Alexa 647 fluorescence are perfectly separated by the optical setup of a flow cytometer, since the corresponding excitation and emission wavelengths are very different. Cells were allowed to internalize fluorescent dextrans for 20 minutes, then washed and incubated further in normal HL5 medium. At the indicated times, an aliquot of cells was analyzed by flow cytometry (Fig. 2A). The fluorescence emitted by Oregon green was minimal at time 0, indicating a very acidic pH (see below), then gradually increased during the chase, indicating that the fluid phase was transferred to a less acidic compartment. After 45 minutes, Oregon green fluorescence gradually decreased, with a kinetic identical to that observed with the pH-insensitive Alexa 647, and similar to that reported previously for the recycling of fluid phase to the extracellular medium [3]. To determine the endosomal pH at each time point, we calculated the ratio of Oregon green and Alexa 647 fluorescence (Fig. 2B) and compared it to a calibration curve obtained in the same experiment as described above (Fig. 2B inset). These results indicated that the pH of early endocytic compartments (lysosomes) was very acidic (pH<3.5, see Discussion). The highest pH values observed during the chase were between 4 and 5, representing a minimal value for the pH of late endosomal compartments (see Discussion).

We considered the possibility that selective loss of Oregon green in endosomal compartments could bias the observations. To test this, cells having endocytosed dextrans (20 minutes) were chased for 0 to 60 minutes. They were then transferred to neutral HL5 (pH 7.4) supplemented with NH_4_Cl and sodium azide to neutralize endocytic compartments. This allowed us to measure the total Oregon green fluorescence irrespective of the original pH of the endocytic compartments. The total Oregon green/Alexa 647 ratio was identical at all times (data not shown) indicating that Oregon green was not selectively degraded or altered in endocytic compartments.

### Single-cell pH determination

**Figure 3:**
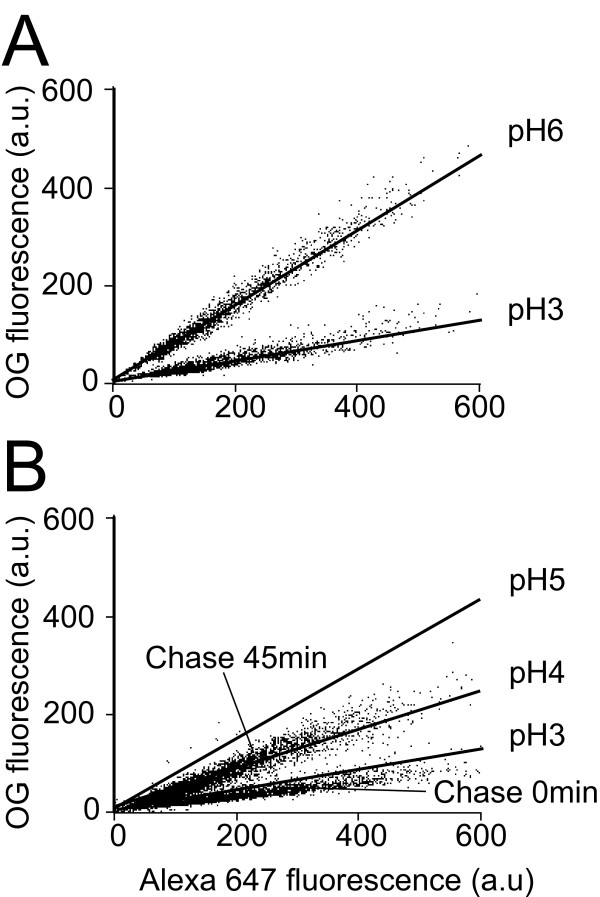
**Single-cell endosomal pH determination**. (A) To calibrate this experiment, cells having internalized a mixture of fluorescent dextrans were incubated at a defined pH in the presence of sodium azide and ammonium chloride, and analyzed by flow cytometry as described in Figure 2. Each dot represents a single cell with the indicated fluorescence values. For comparison, cells incubated at pH3 and pH6 were both represented in one single graph. The lines drawn separate in two equal parts each population of cells. (B) Cells were allowed to internalize fluorescent dextrans, then further incubated in medium for either 0 minutes or 45 minutes. While the amount of endocytosed dextran was extremely variable from cell to cell, the fluorescence ratio, indicating endosomal pH, was remarkably conserved.

One of the advantages of flow cytometry is that it allows single-cell measurements. This is shown in Fig 3, where the Oregon green and Alexa 647 fluorescence of each cell is indicated. After 20 minutes of internalization (time 0), the total fluorescence level was very variable from cell to cell (Fig. 3B), indicating huge cell-to-cell variability in the amount of internalized fluid phase. However the fluorescence ratio was remarkably constant (Fig. 3B), indicating that the lysosomal pH was similar in every cells. With further chase, endosomal pH rose and was higher than 4 in most cells after 45 minutes of chase (Fig. 3B).

## Discussion

In this study, we describe a new method for measuring endosomal pH in *Dictyostelium *amoebae. This method makes use of two dextran-coupled fluorophores, one pH sensitive, and the other one not. The pKa of the pH-sensitive probe (Oregon green) proved to be more adequate for measuring the very acidic pH observed in *Dictyostelium *lysosomes. In addition, while endosomal pH values in *Dictyostelium *were usually determined by measuring the total fluorescence of a population of cells in a spectrofluorimeter, we used a flow cytometer. From a practical point of view, this makes use of small amounts (250 μg/ml) of commercially available reagents and it requires only few cells (a few thousand for each time point). These are serious practical improvements compared to the use of a classical spectrofluorimeter, where many more cells (usually at least 10^6^) and much more fluorescent molecules (up to 20 mg/ml) were necessary to obtain a sufficient signal. This might also be critical in some situations, since in our hands lysosomal acidification appeared less efficient at concentrations above 5 × 10^6 ^cells/ml (data not shown). Finally, a flow cytometer only measures cell-associated fluorescence, and can thus be used while cells are still in HL5 medium [14], despite its autofluorescence. On the contrary, cells are frequently washed with a low-fluorescence buffer (phosphate buffer) when using a spectrofluorimeter, and this could induce changes in endosomal pH. All these considerations might account for the small quantitative differences between our results and the results published previously.

Qualitatively, our results are in agreement with previous observations, indicating that internalized fluid phase is very rapidly found in acidic compartments (lysosomes), then gradually transferred to less acidic compartments (post-lysosomes) after 30 minutes (20 minutes endocytosis + 10 minutes chase). Our results add the surprising notion that the pH in lysosomal compartments is extremely acidic, since we observed a total extinction of Oregon green fluorescence. Strictly speaking, these results indicate a lysosomal pH below 3. However, since Oregon green fluorescence is minimal below pH 3.5, it seems only safe to assume that lysosomal pH is lower than 3.5 in *Dictyostelium*. This is not contradictory with previous results, which observed total protonation of FITC-dextran (pH<5) [4] and of aminomethylphosphonate (pH<4.3) [8] in lysosomes. Although this value might seem extremely low, we have observed that *Dictyostelium *lysosomal enzymes can function in a broad range of pH, from 3 to 6 (data not shown), suggesting that they could indeed be functional in such unusually acidic conditions. Note that the relatively long pulse (20 minutes) did not allow us to follow the rapid acidification of lysosomes, but it was preferred to a shorter pulse because the internalized signal was higher. Very short pulses (down to 1 minute) revealed as expected partial acidification at earlier times but did not modify significantly the results at longer chase times (data not shown).

As expected, the method presented here also detects the existence of a less acidic post-lysosomal compartment [3], since upon prolonged chase the pH of endosomal compartments increased to values above 4. These values represent a minimum for the pH of late endosomal compartments, since the measured pH corresponds to the average of all cellular compartments in which dextran is found at any given time. Given the relatively slow recycling rate of internalized fluid phase (t1/2> 90 minutes: 20 minutes endocytosis + 70 minutes chase), and the relatively small number of post-lysosomal compartments compared to lysosomes [6], it seems likely that at any given time only a fraction of the endocytosed fluid phase is present in post-lysosomes, while a significant portion is still present in acidic lysosomes. In other words, the method described here is suitable for measuring the pH of early endosomal compartments (lysosomes), or for defining qualitative variations in endosomal pH, but it is not ideal for the precise determination of post-lysosomal pH.

The main advantage of the technique presented in this report is its simplicity. It should allow researchers studying the *Dictyostelium *endocytic pathway to determine endosome dynamics (endocytosis of fluid phase, transfer from lysosomes to post-lysosomes, recycling) as well as the pH of the various endocytic compartments. It is thus particularly suited to detect phenotypic changes in the endosomal pathway of *Dictyostelium *mutants.

## Competing interests

The authors declare that they have no competing interests.

## Authors' contributions

AM, PC and EL conceived and designed experiments, and analyzed results. AM performed experiments. PC drafted the manuscript. All authors read and approved the final manuscript.

## Supplementary Material

Additional file 1 **pH-dependent fluorescence of FITC, Oregon green, and Alexa 647**. FITC-dextran, Oregon green-dextran, or Alexa 647-dextran were added to HL5 medium at the indicated pH. The fluorescence was measured in a spectrofluorimeter and expressed as a percentage of the maximal value for each fluorophore. Fluorescence decreased at acidic pH for FITC and Oregon green, but not for Alexa 647. Click here for file
